# DIGITALIZATION OF HEALTH SERVICES FOR OLDER ADULTS: HEALTH INEQUALITIES AND THE COVID-19 PANDEMIC

**DOI:** 10.1093/geroni/igad104.1796

**Published:** 2023-12-21

**Authors:** Alex Hall, Charlotte Eost-Telling, Fiona Beyer, Abodunrin Aminu, David Sinclair, Dawn Craig, Barbara Hanratty, Chris Todd

**Affiliations:** University of Manchester, Manchester, England, United Kingdom; National Institute for Health and Care Research (NIHR) Older People and Frailty Policy Research Unit, Manchester, England, United Kingdom; National Institute for Health and Care Research (NIHR) Older People and Frailty Policy Research Unit, Newcastle-upon-Tyne, England, United Kingdom; National Institute for Health and Care Research (NIHR) Older People and Frailty Policy Research Unit, Manchester, England, United Kingdom; Newcastle University, Newcastle upon Tyne, England, United Kingdom; National Institute for Health and Care Research (NIHR) Older People and Frailty Policy Research Unit, Newcastle-upon-Tyne, England, United Kingdom; Newcastle University, Newcastle upon Tyne, England, United Kingdom; National Institute for Health and Care Research (NIHR) Older People and Frailty Policy Research Unit, Manchester, England, United Kingdom

## Abstract

There is a growing role for digital technologies in society, but concerns that older adults may be disadvantaged and excluded with the growth of use of these technologies. The COVID-19 pandemic led governments across the world to mandate lockdowns and social restrictions. This was accompanied by rapid implementation of many digitalized services, including to access health and social care. This paper reports on a project to advance our understanding of how health and social care services for older adults (65+) have been digitalized during the COVID-19 pandemic, and to learn what has worked well and what has not, with a specific lens on health inequalities. We report main findings from three workstreams: (i) a mapping review, to identify (a) the types of evidence available on the digitalization of health and social care services for older adults during the COVID-19 pandemic, and (b) the extent to which factors related to health inequalities have been considered in this evidence; (ii) quantitative analysis of health care records to examine the impact of digitalization on access to primary care for older people in England during the COVID-19 pandemic, with a focus on incidence rate of consultation types (face-to-face, telephone and video) examined against age, gender, ethnicity and socioeconomic status; (iii) qualitative work to explore the experiences of UK older adults of South Asian and Black African or Caribbean descent of using digital technologies to access primary care. We consider how digitalization of health and social care services relates to health inequalities for older people.

